# Transcription Factor-Based Differentiation of Pluripotent Stem Cells: Overcoming the Traps of Random Neuronal Fate

**DOI:** 10.3390/biomedicines13112783

**Published:** 2025-11-14

**Authors:** Georgie McDaid, Jaime Vanek, Brett Cromer, Huseyin Sumer

**Affiliations:** Department of Chemistry and Biotechnology, Swinburne University of Technology, Melbourne, VIC 3122, Australia; gmcdaid@swin.edu.au (G.M.);

**Keywords:** neurons, glia, transcription factors, cell differentiation, pluripotent stem cells, neuronal differentiation, neurodegeneration

## Abstract

Developing robust methods to differentiate pluripotent stem cells (PSCs) into specific neuronal subtypes is crucial for advancing neuroscience research, including disease modelling and regenerative medicine. Research in this area has primarily focused on generating and studying excitatory neurons, often in co-culture with primary astrocytes to support maturation. Due to the shared ectodermal lineage of these cell types, any mesoderm derived cells, such as microglia, are absent using traditional methods of culture. To more accurately model the intricate complexity of the brain and its normal neuronal physiology, it is important to incorporate other critical neural subtypes, such as inhibitory interneurons and various glial cells. This review highlights recent progress in using transcription factor-based in vitro differentiation strategies to generate these diverse neural populations. A major advantage of this approach is the ability to rapidly produce highly specific cell types in a controlled manner, allowing for the precise seeding of cells at defined anatomical and physiological ratios. This controlled methodology enables the creation of more accurate and reproducible in vitro models, including two-dimensional (2D) and three-dimensional (3D) cultures and organoids, thereby moving beyond the limitations of random differentiation from neuronal progenitor cells. Despite these advances, key challenges remain, including reproducibility between pluripotent stem cell lines, off-target transcriptional effects of exogenous factors, and incomplete phenotypic maturation of derived cells. Addressing these constraints is essential for translating transcription factor-based approaches into robust and clinically relevant neural models.

## 1. Introduction

Pluripotent stem cell (PSC)-based technologies have transformed neuroscience research by providing an infinite source of human cells for disease modelling, drug discovery, and regenerative applications. The ability to differentiate PSCs into defined neuronal subtypes has created opportunities to study human neural development and pathology in ways that were not previously possible with animal models or post-mortem human tissue. Over the past decade, major progress has been made in generating excitatory neurons from PSCs. These cells have been widely utilised to investigate cortical function, synaptic connectivity, and excitotoxic mechanisms relevant to neurodegenerative diseases. In most instances, these excitatory neurons are co-cultured with primary rodent or human astrocytes to promote synaptic maturation.

Despite these advances, excitatory neurons alone represent only one component of the brain’s highly complex cellular network. To fully capture the cellular diversity and intricate interactions that underlie both normal physiology and disease pathogenesis, it is essential to expand differentiation strategies to encompass additional neuronal subtypes and glial populations. Inhibitory interneurons, for example, play critical roles in maintaining excitatory-inhibitory balance, shaping network oscillations, and preventing hyperexcitability. Disruption of inhibitory signalling is a hallmark of numerous neurological disorders, including some epilepsies. Similarly, glial cells—including astrocytes, microglia, and oligodendrocytes—are now recognised not merely as supportive elements, but as active participants in synaptic regulation, neuroinflammation, and metabolic homeostasis. Dysregulation of glial function is increasingly implicated in the initiation and progression of neurodegenerative diseases such as Alzheimer’s disease, Parkinson’s disease, and amyotrophic lateral sclerosis.

Developing protocols that generate these diverse neuronal and glial populations from PSCs therefore represents a critical step toward creating more physiologically relevant in vitro models. Traditional approaches, which often rely on spontaneous or growth factor-driven differentiation from neuronal progenitors, tend to produce heterogeneous cell mixtures with variable proportions of cell types. To generate guided brain organoids, human PSCs are cultured in embryoid bodies, which are directed toward an ectodermal lineage. Over the course of organoid maturation, neural progenitor cells (NPCs) give rise to a broad spectrum of neuronal and glial populations. However, as they originate exclusively from ectodermal tissue, these brain organoids lack non-ectodermal elements, which includes important microglia and vascular components [1].

While valuable, these methods limit reproducibility and complicate the study of cell type-specific mechanisms. In contrast, transcription factor-driven differentiation strategies provide a more controlled and efficient approach to generate a specific cell type. By introducing lineage-specific transcription factors, researchers can rapidly and reproducibly generate specific neuronal and glial subtypes. Importantly, this method enables the seeding of defined cell types in co-culture at precise ratios, better reflecting the cellular architecture of the brain. This precision is especially advantageous when modelling diseases in which the relative abundance of particular cell types, or their interactions, are central to pathology.

While conventional growth factor-driven differentiation from NPCs has been instrumental in generating neuronal and glial populations, these approaches often yield heterogeneous cell populations that require additional purification steps such as FACS. Transcription factor (TF)-based strategies, by contrast, enable lineage commitment through the direct activation of gene regulatory programs, often resulting in faster and more synchronised differentiation. This review critically examines how TF-mediated differentiation compares with other emerging modalities such as CRISPRa-mediated transcriptional activation and small-molecule induction, with particular focus on their reproducibility, efficiency, and translational potential.

## 2. Neurons of the Central Nervous System

Neurons are the fundamental signaling units of the central nervous system (CNS), forming complex circuits that underlie behaviour and cognition. Their diversity in structure, neurotransmitter identity, and function provides both an opportunity and a challenge for in vitro modelling. Pre-synaptic neurons can be broadly classified into two functional categories, excitatory and inhibitory, based on the type of neurotransmitter they release and their effects on post-synaptic targets. Although some neurotransmitters can exert both excitatory and inhibitory effects dependent on the post-synaptic receptor type, excitatory neurons primarily release glutamate, but may release other excitatory neurotransmitters such as acetylcholine and serotonin [2]. Inhibitory neurons primarily release gamma-aminobutyric acid (GABA), but may release glycine, particularly in the spinal cord [2]. In the CNS, excitatory and inhibitory neurons are primarily referred to as glutamatergic or GABAergic neurons, respectively, based on their predominant neurotransmitter. This classification simplifies the vast array of functional roles and interactions present within neural circuits.

Neurons comprise roughly half of the total number of cells in the CNS. Roughly 80% of cortical neurons are excitatory (primarily glutamatergic pyramidal cells), while about 20% are inhibitory interneurons (mostly GABAergic) [3], a ratio that helps maintain the balance between signal propagation and suppression to prevent runaway excitation.

### 2.1. Excitatory Glutamatergic Neurons

Glutamatergic neurons are the predominant excitatory cells of the cerebral cortex and hippocampus, responsible for information transmission and plasticity. In vitro, their generation from PSCs enables reproducible access to cortical-like excitatory populations that display pyramidal morphology, synaptic connectivity, and functional glutamatergic signaling. Dysfunction in glutamatergic signalling or excitatory neuron activity is linked to neurological disorders such as epilepsy [4], and neurodegenerative diseases like Alzheimer’s disease [5]. Glutamate is a neurotransmitter that typically functions to stimulate neurons by depolarisation but also controls the differentiation of oligodendrocyte precursor cells (OPCs) into oligodendrocytes (OLs). High levels of glutamate or over-expression of glutamate receptor genes can act as an excitotoxin, overstimulating cells and become highly toxic due to excessive depolarisation and Ca^2+^ entry, which can lead to cell death in both neurons and OLs [6]. Not only can direct damage of OLs occur, but because of damaged OLs, the functioning of other cells including neurons, astrocytes and microglia can be impaired too, which further disrupts the signalling between the CNS and other cells of the body.

### 2.2. Inhibitory GABAergic Neurons

GABAergic interneurons provide inhibitory control that maintains excitatory-inhibitory balance within neuronal circuits. When GABAergic neurons are dysfunctional, the resulting imbalance contributes to a wide array of neurological and psychiatric disorders. In epilepsy, impaired GABAergic interneuron development or migration may lead to failure of network inhibition, excessive excitation and enhanced seizure susceptibility. Recent cerebral organoid studies have shown that genetic neurological disorders, including Parkinson’s disease, can alter the excitatory-to-inhibitory balance, underscoring the contribution of interneuron dysfunction to cortical hyperexcitability and disease [7].

### 2.3. Other Neuronal Subtypes

Beyond glutamatergic and GABAergic neurons, several specialised neuronal subtypes shape CNS circuitry and disease vulnerability. Cholinergic neurons regulate attention and memory and are profoundly affected in Alzheimer’s disease [8]. Dopaminergic neurons modulate reward and motor control and are the primary targets of degeneration in Parkinson’s disease [9]. Serotonergic and histaminergic neurons, though relatively few in number, influence arousal, mood, and circadian rhythms [10]. Understanding how TF-based differentiation can reproducibly generate these distinct populations of neurons, through TFs such as Ascl1, Nurr1, and Lmx1a [11], remains a crucial challenge for constructing multicellular models of brain function.

## 3. Glial Cells and Their Role in the CNS

Glial cells, or neuroglia, refer to the non-neuronal cells within the CNS. Neuroglia have a key role in responding to neurological injury, regulating synaptic activity, and preserving neural homeostasis. Glial cells have a dual function in neurodegenerative diseases, such as multiple sclerosis (MS), Parkinson′s disease (PD), and Alzheimer′s disease (AD), as they can both promote and prevent neurodegeneration. In other CNS injuries, such as during a stroke, glial cells contribute by regulating inflammation, clearing cellular debris, releasing neuroprotective or neurotoxic factors, and influencing repair processes such as scar formation and synaptic remodelling [12]. Despite their significance, the roles of glial cells in contributing to such disorders is relatively poorly understood. This is due, in part, to the lack of sufficient tools for efficient generation of in vitro glial cell models.

In the CNS, neurons and glial cells are present in roughly comparable numbers, with glia constituting about half of all cells, though their proportions vary by brain region and developmental stage. Across the regions of the brain, astrocytes account for about 20–40% of glia; oligodendrocytes make up around 45–75%; and microglia represent about 5–10% [13,14]. Together, these neuronal and glial ratios ensure a tightly regulated environment where excitation and inhibition are balanced and neural circuits function efficiently.

Emerging TF-based reprogramming strategies now permit targeted generation of astrocytes (SOX9, NFIB) [15], oligodendrocytes (SOX10, OLIG2, NKX6.2) [16], and microglia (SPI1, CEBPA) [17], allowing systematic in vitro study of the individual and cooperative functions of these cells in processes such as neuroinflammation and synaptic regulation.

### 3.1. Astrocytes

Astrocytes are recognised as active participants in a wide range of physiological processes. Astrocytes have distinct morphological features that distinguish them from other glial and neuronal cells; they feature a star-like morphology and have multiple fine processes that can contact thousands of neuronal synapses [18]. Their intricate interactions with neurons and other glial cells underscore their significance in both healthy brain function, and the progression of neurological disease. They play a multifaceted role in essential brain function, including structural support, ion homeostasis, neurotransmitter recycling, synaptic transmission regulation, defence against oxidative stress, energy storage, and tissue repair [19]. Currently, many of astrocytes’ functions are largely understood from mouse models, which are significantly less complex than human astrocytes [20].

**Figure 1:**
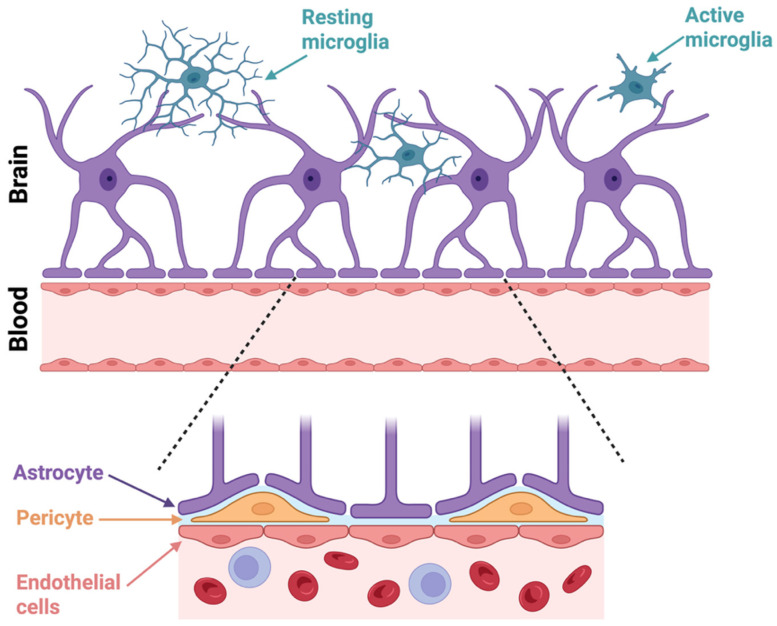
Cellular interactions at the blood–brain barrier (BBB). In the upper panel, astrocytes (purple) extend their end-feet processes toward the vasculature, which forms a key structural component of the BBB. Resting microglia (blue) with ramified processes are depicted patrolling the brain parenchyma, while active microglia (blue) exhibit a more amoeboid morphology associated with immune activation. The lower magnified panel shows the BBB in greater detail, highlighting the interactions between astrocyte end-feet, pericytes (orange), and endothelial cells (pink). Endothelial cells form the inner lining of brain capillaries and are tightly connected by junctions that regulate selective permeability. Pericytes are embedded in the basement membrane and contribute to vascular stability and BBB maintenance. This cellular architecture is critical for maintaining brain homeostasis and the regulation of substances between the bloodstream and the CNS. Created in BioRender. Mcdaid, G. (2025) https://BioRender.com/gfnupjd (accessed 1 October 2025).

Along with microglia, they contribute to the brain’s immune response, and aid in repairing nervous tissue after injury [21]. This response, astrogliosis, is implicated in neurodegenerative diseases such as AD and PD, and plays a crucial role in both the development and prevention of neurological damage. Reactive astrocytes can release a wide variety of extracellular signalling molecules, including inflammatory modulators, chemokines, cytokines, along with various neurotrophic factors [22]. Some of these factors can be neuroprotective (IL-6 and TGF-β) or neurotoxic (IL-1β and TNF-α). Perhaps the most important role of astrocytes is their tight regulation of the blood–brain barrier (BBB), by surrounding the blood vessels with their end-feet and releasing signals that strengthen tight junctions between endothelial cells (Figure 1) which are critical for the selective permeability of the BBB [23]. Hence, they regulate barrier integrity, blood flow, and ion balance, and their dysfunction can lead to BBB breakdown in neurological disease [24].

### 3.2. Oligodendrocytes

Oligodendrocytes maintain integrity of the CNS by forming and sustaining the myelin sheath around neuronal axons. Dysfunction of OLs is central to several neurological diseases, most notably MS [25]. Emerging research also implicates OL abnormalities in conditions like AD [26], highlighting their broader significance in brain health and disease. Myelin ensheathes nerve axons in multiple layers of lipid-rich membranes that wrap around the axolemma between the nodes of Ranvier, forming a stable and protective coating. Composed of roughly 70–80% lipids, myelin enables saltatory conduction, allowing rapid propagation of electrical impulses throughout the CNS [27]. By insulating axons, it enhances both the speed and efficiency of signal transmission, facilitating effective neuronal communication across the CNS. Nevertheless, because the axolemma is difficult to observe directly, the precise mechanisms by which myelination accelerates action potential propagation remain incompletely understood.

Defined features of mature OLs include their small cellular bodies that contain nuclei with significant amounts of chromatin as well as multiple fine cellular extensions containing cytoplasmic granules [27]. Thus, myelin is crucial for movement and cognition functioning which includes learning and memory [28]. The myelin sheath is non-continuous and segmented, as there is a gap between each membrane (nodes of Ranvier) and terminates at the paranodal loops. The electrical signal ‘jumps’ from one node to the neighbouring node. In doing so, the velocity of conductance of nerve is increased and the energy required to transmit this signal is reduced [29].

OLs also have a variety of other important functions and roles within the CNS. They encourage congregation of sodium channels along the axon to allow saltatory action potential propagation across internodes in nerves, as well as maintenance of voltage-gated ion channels and action potential [30]. Furthermore, they provide trophic support for neurons by creating factors such as glial cell line derived neurotrophic factor (GNDF), brain derived neurotrophic factor (BDNF) or insulin-like growth factor (IGF-1). OLs also provide metabolic support to axons and aid in multiple forms of neuroplasticity [31].

### 3.3. Microglia

Microglia are the only innate immune cell that reside in the CNS [32]. Unlike other glial and neural cells which arise from neuroectodermal origin, microglia originate from mesodermal haemopoietic origin [33]. In the early phases of embryogenesis, microglia are produced from myeloid precursors in the yolk sac and travel to the CNS prior to the early closing of the blood–brain barrier (BBB) [34,35]. Microglia maintain a stable population within the CNS throughout the lifespan by self-renewal [36,37].

**Figure 2:**
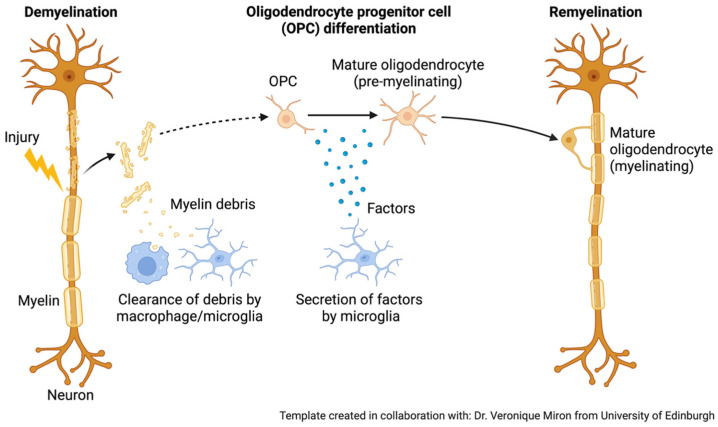
Role of microglia and oligodendrocytes in remyelination after damage. During remyelination, microglia play a crucial role by releasing signalling molecules that create a supportive environment for repair. After clearing myelin debris, these immune cells secrete factors that encourage oligodendrocyte progenitor cells (OPCs) to survive, proliferate, and differentiate into mature oligodendrocytes. This microglia-driven communication is essential for promoting the regeneration of myelin and restoring normal neuronal function [40]. Created in BioRender. Mcdaid, G. (2025) https://BioRender.com/f4vl6r9. Adapted from “Remyelination.” retrieved from https://app.biorender.com/biorender-templates (accessed 28 September 2025).

In addition to innate phagocytotic activity, microglia play a crucial role in regulating brain homeostasis by controlling neurogenesis, oligodendrogenesis, and facilitating neuronal survival. Synaptic pruning by microglia is a critical developmental process in which these immune cells refine neural circuits by eliminating excess or weak synapses, ensuring efficient brain connectivity. In adulthood, however, microglial activation extends beyond pruning and plays a central role in responding to injury, infection, or pathological protein accumulation. By phagocytosing neural precursor cells, microglia are known to suppress the growth of cortical neurons [38]. Microglia release signalling molecules that can be inflammatory and cytotoxic to oligodendrocytes, but others that are essential to oligodendrogenesis and myelination [39,40] (Figure 2). Additionally, microglia phagocytose OPCs during early postnatal development to regulate myelination [41].

Alteration of neurogenesis is implicated in many brain pathologies, including neurodegenerative diseases [42]. Impairment of the normal structure of microglia can be caused by cell senescence during ageing, by intense inflammation following infections or disease, or from chronic stress exposure. As such, neurological diseases such as AD, PD, amyotrophic lateral sclerosis, depression, and schizophrenia are all hypothesised to be at least partly of microglial origin [43,44,45]. Microglia interact closely with astrocytes, pericytes and endothelial cells at the blood–brain barrier (BBB) (Figure 1). In response to infection, injury, or disease, microglia may become activated, releasing pro-inflammatory chemicals that disrupt endothelial cells and increase BBB permeability.

Activated microglia release cytokines, chemokines, and reactive oxygen species [46], contributing to neuroinflammatory responses that are protective when clearing debris or toxic aggregates but detrimental when chronically sustained, leading to synaptic loss and neuronal dysfunction. This function is critical for CNS defence but can cause BBB collapse in chronic inflammation or neurodegenerative disorders [47]. This dual role is especially evident in neurodegenerative conditions such as AD and PD, where microglia may both limit disease progression by removing harmful proteins and exacerbate pathology through persistent inflammation and aberrant pruning. In the early stages following an ischemic stroke, microglia that adopt an anti-inflammatory state and secrete neurotrophic factors can support repair processes and facilitate neurological recovery at the site of injury. In contrast, during the later stages, a shift in microglia toward a pro-inflammatory phenotype, accompanied by the release of damaging inflammatory mediators, can worsen tissue injury. These dynamics highlight the therapeutic promise of strategies aimed at steering microglial polarisation toward an anti-inflammatory profile to mitigate secondary damage and improve outcomes in ischemic stroke [48]. Thus, microglia serve as both guardians and potential drivers of neurodegeneration, highlighting the need for therapies that modulate their activity toward neuroprotection while minimising harmful consequences.

## 4. Glial Contributions to Neurodegenerative and Epileptic Disorders

Neuronal cells of the CNS do not regenerate following injury caused by illness, ischemia, or trauma. Cells in the brain, generally, are able to live for the entire lifespan. The most common reason for neurodegenerative disease development is that a cell, or multiple cell types, becomes dysfunctional [49].

Prominent neurodegenerative diseases include AD, PD, amyotrophic lateral sclerosis (ALS), frontotemporal dementia (FTD), chronic traumatic encephalopathy (CTE), and multiple sclerosis (MS). Hallmarks of disease include neuronal cell death, pathological protein aggregation, inflammation and synaptic network defects [50]. Neurons are affected by the early symptoms of neuronal pathology in AD, PD, and ALS, and hence these diseases can be considered as ‘primary’ neurodegenerative disorders. In contrast, neurodegeneration and neuronal involvement in MS appears to be subsequent to the processes that attack myelin in the CNS [51]. Neuroinflammation, including astrogliosis and microgliosis, is a key characterising feature of neurodegenerative disorders [52,53]. Progressive accumulation of protein aggregates, such as β-amyloid, tau and α-synuclein, is a hallmark of neurodegeneration, driving neuronal loss and contributing to disorders like AD and PD [54]. Neuronal stress then induces microglial activation that enhances the production of both pro- and anti-inflammatory substances, as well as phagocytic activities to clear neuronal debris [55].

In vitro studies that mimic the complex brain environment and include glial cells such as astrocytes and microglia are essential for a better understanding of the progression of neurodegenerative diseases and dissecting the role of glia as a protector or catalyst in neurodegeneration. The TREM2 receptor in microglia has been highlighted as a key modulator of neuroinflammatory responses in AD [56], suggesting that targeting this pathway could afford therapeutic benefits.

Active demyelination, and active tissue damage in general, is associated with microglial and astrocyte activation [53]. Astrocytes are implicated in neurodegenerative diseases like AD and PD, and they are involved in development and prevention of damage during strokes [12]. Astrocytic cells are also known to swell with fluid during the day, which they then remove at night, clearing the brain of toxins and other waste material [19].

OLs and the myelin sheath of axons are highly susceptible to damage, more so than other molecules and cell types of the nervous system such as neurons, astrocytes and microglia [31]. Due to the high metabolic activity of OLs, they consume a significant amount of oxygen and ATP, producing hydrogen peroxide as a byproduct. This is toxic in high concentrations and can create reactive oxygen species if it is not metabolized adequately. Moreover, as production of myelin is controlled by myelin synthetic enzymes, which require iron as a cofactor, causing a high intracellular concentration of iron. Low amounts of glutathione, an antioxidant, are present so the iron required as a cofactor can cause free radical formation and lipid peroxidation. Hence, myelin and OLs are highly vulnerable to oxidative damage [57].

Moreover, inflammatory cytokines can cause damage to OLs. This includes tumour necrosis factor alpha (TNFa), which induces apoptosis by binding to the p55 TNFa receptor or interferon gamma (IFNy), a process that is highly toxic for proliferating OPCs and mildly toxic for immature OLs [30]. Inflammatory mediators indirectly affect OLs by stimulating free radical production impacting microglia and astrocytes. This occurs due to excess oxygen and nitric oxide, which are highly toxic to mitochondria because they block proteins essential in the respiratory chain causing mitochondrial damage [31].

The manifestation of diseases and symptoms associated with demyelination varies widely, as each demyelinating event is unique and influenced by numerous factors. Demyelination is most prominently observed in MS, where the immune system mistakenly attacks the myelin sheath, resulting in scarring and lesion formation. This immune-mediated damage leads to the breakdown of myelin, exposing and degenerating the underlying axons over time, ultimately causing neuron death [31]. Consequently, neuronal dysfunction occurs, impairing both brain and cognitive function.

The “tripartite synapse” refers to a synaptic arrangement in which communication occurs not only between the pre- and post-synaptic neuronal compartments, but also involves surrounding astrocytic processes, which closely envelop the synapse to regulate neurotransmitter clearance, ion balance, and modulatory signalling [58]. Dysfunction of the tripartite synapse is highly relevant in epilepsy, as impaired astrocytic regulation of neurotransmitter clearance and ion homeostasis can lead to heightened neuronal excitability and the uncontrolled network synchronisation that drives seizure activity [59]. Adding an additional layer of complexity, OPCs are now recognised as highly interactive synaptic partners as they form direct glutamatergic and GABAergic contacts with neurons, exhibit calcium transients in response to synaptic activity, release paracrine signals that regulate neuronal excitability, and can modulate myelination to match activity patterns [60]. As an alternative, the “penta-partite synapse” is an advanced conceptual model that expands the traditional view of synaptic structure and function to encompass five dynamically interacting cellular elements: the pre- and post-synaptic neuronal compartments, astrocytes [58], microglia [61], and OPCs [60]. Together, these five elements create a highly dynamic, multicellular signalling network in which neuronal transmission is continuously shaped by glial surveillance, immune signalling, and progenitor cell plasticity.

## 5. Building the Diseased Brain in Dish

As increasing evidence suggests that glial cells are implicated in numerous neurodegenerative and neurological diseases, including AD, PD, epilepsy and MS, stem cell-based approaches for generating glia offer a valuable platform for studying the cellular mechanisms of such CNS disorders, as well as providing potential avenues for regenerative medicine. Pluripotent stem cells can be directed to differentiate into neurons and glia through carefully timed changes to culture media and a progression through various progenitor states, but this process is often slow and cumbersome. In contrast, advances in transcription factor-based differentiation protocols enable more rapid and efficient generation of specific neural cell types by directly steering pluripotent stem cells toward desired lineages, enabling rapid differentiation in vitro.

Building a penta-partite system in vitro using PSCs involves recreating the complex interactions between excitatory and inhibitory neurons, astrocytes, microglia, and OPCs/OLs in a controlled dish environment. PSCs can be differentiated into excitatory and inhibitory neurons that form functional pre- and postsynaptic networks, establishing the foundation of the synapse. Astrocytes also derived from PSCs can be co-cultured to support synaptic maturation, regulate neurotransmitter clearance, and modulate gliotransmission. Microglia are incorporated to mimic immune surveillance, synaptic pruning, and cytokine-mediated modulation of neuronal activity, while OPCs are added to replicate progenitor-neuron interactions, calcium signalling, and activity-dependent influence on network excitability.

Emerging advances in disease modelling using stem cells have paved the way for new research into specific therapeutic treatments targeting glial cells [62,63,64]. Recent efforts to speed up the differentiation process by using TF-based differentiation have shortened the timeframe to produce functional cells. Cell fates are determined by TF regulation during embryonic development. Ectopic expression of various TF combinations has been demonstrated to quickly reprogram the cell type in vitro. Reprogramming of somatic, differentiated, fibroblast cells back to a pluripotent state by overexpression of transcription factors such as Oct4, Klf4, c-Myc and Sox2 has been described by Takahashi et al. [65]. Differentiation using transcription factors leads to more rapid differentiation, because they bypass the more complex signalling pathways triggered by growth factors, taking the differentiation process from many months to just a few weeks. The most common source of PSCs are embryonic stem cells (ESCs); however, they pose many ethical concerns as they are sourced from the inner cell mass of the blastocyst which generally leads to the eradication of the embryo [66]. As an alternative to negate these ethical concerns, induced PSCs by reprogramming somatic cells to a pluripotent state [65] can be used.

Reprogrammed neurons and glia have become powerful tools for reproducing disease-specific cellular phenotypes. Ngn2-induced differentiation of patient-derived induced pluripotent stem cells to model PCDH19-clustering epilepsy (PCDH19-CE) produced functional neurons rapidly, revealing that mutant neurons showed accelerated maturation and increased dendritic arborisation, while mosaic cultures exhibited enhanced action potential firing and hyperexcitability [67]. As an in vitro epilepsy model, this system effectively recapitulates key neurophysiological features of PCDH19-CE, including neuronal hyperexcitability and network dysfunction. These findings indicate that PCDH19 mutations disrupt normal neuronal development and excitability, and that Ngn2 hiPSC-derived mosaic neurons provide a powerful in vitro model for studying disease mechanisms and testing targeted therapies for PCDH19-CE. As a model of Parkinson’s disease (PD), ASCL1, PITX3, NURR1, and LMX1A-induced dopaminergic neurons show α-synuclein accumulation as seen in PD [68], offering a physiologically relevant in vitro model of the ageing brain.

In more recent reprogramming methods, direct neuronal conversion has emerged as a powerful strategy for modelling age-associated neurodegenerative disorders in vitro. By use of brain-enriched microRNAs (miR-9/9* and miR-124) in combination with neuronal TFs NEUROD2 and MYT1L, fibroblasts from individuals with late-onset AD can be efficiently reprogrammed into cortical neurons within a 3D culture environment [69]. This approach preserves donor-specific age signatures, allowing the resulting neurons to mimic key neuropathological features of late onset AD, including extracellular amyloid-β deposition, tau aggregation, dystrophic neurite formation, and neuronal loss. Importantly, early inhibition of amyloid precursor protein processing during reprogramming attenuated these pathological phenotypes, highlighting the system’s relevance for therapeutic testing. Collectively, miRNA-mediated direct neuronal reprogramming provides a robust “disease-in-a-dish” model that captures the cellular aging context critical for understanding and targeting late-onset AD.

In efforts to model the cellular pathology of primary progressive MS, García-León et al. [70] developed a human oligodendrocyte system derived from patient-specific iPSCs. Although the study did not extend to detailed pathological analyses, it demonstrated a robust and efficient protocol for generating high yields of human oligodendrocytes from primary progressive MS donors, establishing a valuable platform for modelling disease-specific mechanisms in progressive MS by introducing SOX10 via lentiviral transduction. By 22 days after transduction, both patient-derived and healthy control lines produced comparable proportions of oligodendrocyte lineage cells, approximately 50–65% O4+ and 10% MBP+, exhibiting similar morphologies and expression profiles for mid-to-late-stage oligodendrocyte markers.

The incorporation of transcription factor- and microRNA-based reprogramming strategies has further accelerated the generation of functional, disease-relevant cell types, enabling rapid exploration of disease mechanisms and therapeutic testing. Importantly, patient-derived induced pluripotent stem cells allow faithful modelling of genetic and age-related disease phenotypes while addressing ethical concerns associated with embryonic sources. Together, these innovations mark a paradigm shift toward more physiologically relevant, patient-specific “disease-in-a-dish” models that bridge the gap between basic research and clinical translation in the study of CNS disorders.

## 6. Differentiation Methods

Efforts to generate glial cells from human PSCs through media supplementation with growth factors and cytokines is an extremely time-consuming process, involving numerous laborious steps. Growth factors are secreted proteins that bind to receptors on the cell to influence cell growth. Signal transduction is the broad term for the act of delivering an external chemical signal to a cell to cause a biological response. When binding to cell surface receptors, signals are sent to other intracellular components, which alters the expression of certain genes within the cell [71]. Cytokines are small peptides that can be classified as growth factors. All cytokines influence signal transduction pathways, but only cytokines that have an impact on the signalling pathways involved in cell growth and differentiation are regarded as growth factors [71]. Commonly used growth factors for neuronal and glial cell development include the neurotrophins, nerve growth factor (NGF), epidermal growth factor (EGF), fibroblast growth factor (FGF), the transforming growth factor β (TGF-β) family [72] and cytokines such as tumour necrosis factor-alpha (TNF-α) and the interleukin (IL) family [72,73]. The successful use of these factors requires precise timing of their addition and removal, as cells must progress through distinct stages of differentiation in a coordinated manner. In contrast, TF-driven differentiation progresses at a significantly faster rate, enabling cells to commit to specific lineages more rapidly and efficiently by directly controlling gene expression rather than relying on extracellular signalling cues.

### 6.1. Neuronal Differentiation

**Figure 3:**
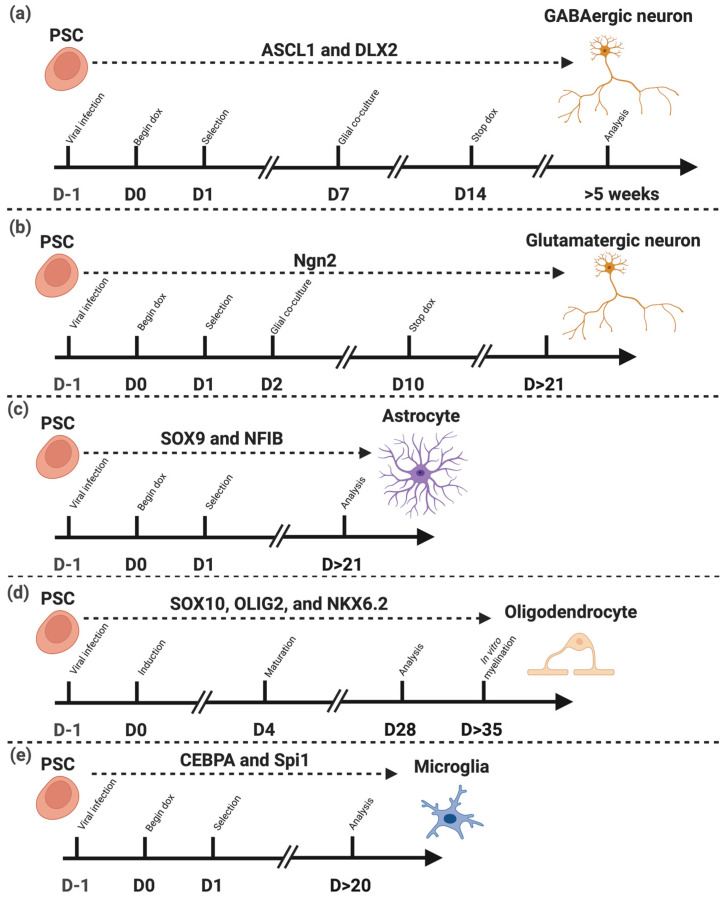
Transcription factor-driven differentiation of pluripotent stem cells (PSCs) into distinct neural cell types. Schematic representation of the differentiation protocols from PSCs into major neural lineages through the forced expression of lineage-specific transcription factors (TFs). (**a**) Expression of ASCL1 and DLX2 directs PSCs toward GABAergic neurons [74]. (**b**) Expression of Ngn2 promotes differentiation into glutamatergic neurons [75]. (**c**) Combined expression of SOX9 and NFIB induces differentiation into astrocytes [15]. (**d**) Co-expression of SOX10, OLIG2, and NKX6.2 drives oligodendrocyte lineage commitment and maturation [16]. (**e**) Expression of CEBPA and Spi1 leads to the generation of microglia [17]. These TF combinations define lineage identity and maturation timing across distinct neural cell types derived from PSCs. **Note:** “D” indicates Day. Dashed lines separate the different cell type differentiation protocols, and arrows indicate the progression of the experimental timeline. Created in BioRender. Mcdaid, G. (2025) https://BioRender.com/jwcbn68 (accessed on 10 November 2025).

Neurons are ectodermal cells, that originate specifically from the neural plate, which undergoes induction and folding to form the neural tube. In more recent neuronal cell differentiation methods, transcription factor-directed excitatory neuronal cells have been differentiated using ASCL1 alone via a lentiviral vector [74]. Zhang et al. [75] produced excitatory neurons from overexpression of Neurogenin-2 or Neurod1 within two weeks using a lentiviral vector (Figure 3). Pure GABAergic neurons have been produced in five weeks by Yang et al. [76] via a lentiviral vector delivering ASCL1 and DLX2 (Figure 3). There are several drawbacks to using lentiviral vectors in therapeutics. Lentiviruses have oncogenic potential, as infected cells may potentially become cancerous through insertional mutagenesis, because viral DNA integrates into the host genome somewhat randomly. This can then cause gene dysregulation by activating oncogenes or inactivating tumour suppressor genes in the host [77]. Lentiviruses, as human pathogens, also have the possibility to unintentionally form a pathogenic virus through recombination [77]. Lentiviral-mediated differentiation approaches, while widely used, often result in variable outcomes due to differences in viral titre, integration efficiency, and copy number across cells [78]. These inconsistencies can complicate downstream analyses and reproducibility. To overcome these limitations, doxycycline-inducible constructs integrated at a single, defined genomic site in all cells offer a more uniform and controllable system, enabling precise temporal regulation of gene expression and more consistent differentiation outcomes.

Doxycycline-inducible expression constructs have been used to generate excitatory neuronal cell lines. These modified cell lines retain their pluripotency but can be directed to differentiate into neurons upon doxycycline addition. While specific neuronal media is still required to support maturation, the cells bypass the conventional stepwise differentiation stages, allowing more direct and efficient neuronal specification. This is due to neurogenin-2, a key transcription factor for neuronal development, being the inducible factor [79,80]. Neurogenin-2 (Ngn2) is a proneural basic helix-loop-helix TF and has been widely employed as a potent driver of excitatory neuronal differentiation from pluripotent stem cells and other progenitor populations, owing to its ability to rapidly activate transcriptional programs associated with cortical glutamatergic neuron identity [81]. Numerous studies have demonstrated that forced expression of Ngn2 can generate neurons with electrophysiological properties and gene expression profiles consistent with excitatory cortical neurons [81], making this approach a valuable platform for disease modelling, functional studies, and high-throughput screening. Nevertheless, despite the strong evidence supporting its role in excitatory fate specification, neuronal populations obtained through Ngn2 induction may not be fully restricted to an excitatory phenotype [80,82]. Instead, Ngn2-induced cells may exhibit features more consistent with a broadly neuronal, but otherwise unspecified, identity. This highlights the importance of carefully validating neuronal identity through molecular, electrophysiological, and functional assays when employing Ngn2-driven protocols, particularly in contexts where precise excitatory lineage commitment is critical for downstream applications.

### 6.2. Astrocyte Differentiation

**Figure 4:**
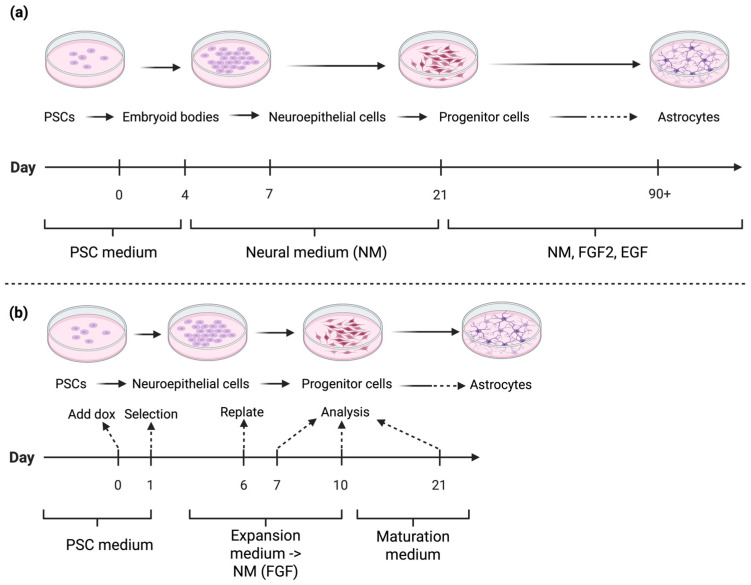
Simplified workflow for in vitro differentiation to astrocytes by: (**a**) Growth factor-mediated differentiation (adapted from methods described by Krencik & Zhang [83] and Mormone et al. [84]). These methods feature long maturation times, and multiple media changes. PSCs are differentiated into embryoid bodies, neuroepithelial cells, and NPCs before maturing into astrocytes. The process involves multiple medium changes and long maturation times. Culture media include PSC medium, neural medium (NM), and NM supplemented with fibroblast growth factor 2 (FGF2) and epidermal growth factor (EGF). (**b**) TF-driven differentiation (Adapted from Canals et al. [15]). PSCs are induced into neuroepithelial and progenitor cells, followed by maturation into astrocytes using doxycycline (dox) induction and selection steps. Media include PSC medium, expansion medium transitioning to NM (with FGF), and maturation medium. **Note:** Solid arrows indicate the sequential progression of differentiation steps. Dashed arrows represent key experimental transitions (e.g., induction, selection, replating, or analysis time points). Created in BioRender. Mcdaid, G. (2025) https://BioRender.com/ipp1ph5 (accessed on 10 November 2025).

Astrocytes are ectodermal cells generated from neural stem cells and radial glial cells in the developing CNS, and they differentiate through a series of signalling pathways and TFs that guide them to mature to support neurons, regulate synaptic activity, and maintain the blood–brain barrier. The astrocyte differentiation protocols described by Krencik & Zhang [83] and Mormone et al. [84] follow similar growth factor routes up to the point of astrocyte maturation. In each case, the process involves carefully timed modifications of the media composition and the sequential application of growth factors, reflecting the dynamic signalling environment necessary for directing precursor cells toward an astrocytic lineage. These iterative changes are essential for evoking developmental cues in vitro, ensuring that NPCs gradually acquire astrocytic characteristics before undergoing terminal maturation. Differentiation using such methods has produced astrocytes within 35 to 120 days (Figure 4) with varying efficiency. Krencik & Zhang [83] produced astrocytes from hPSCs utilising growth factors such as EGF and FGF2, but this process can take over three months to produce ample numbers of astroglial cells, whereas methods such as Mormone et al. [84] reduce the duration of culture to 35 days (Figure 3). Variations in the maturation period is paralleled with the percentage of astrocyte cells expressing glial fibrillary acidic protein (GFAP), which ranged from 55 percent to 90 percent in the two studies [85], indicating a reduction in the efficiency and functionality of the cells produced. Hence, longer maturation culture periods are likely required for physiologically functioning astrocytes using growth factor methods.

Although cellular identity is increasingly understood as being determined by gene regulatory networks driven by TFs, yet multiple TF combinations can yield broadly similar cell types. This redundancy presents both a challenge and an opportunity for cell reprogramming strategies. Transcription factor-based methods for differentiation of astrocytes by Canals et al. [15] produced functional homogenous astrocytes from human ESCs within 21 days, using the overexpression of Sox9 and NFIB. Sox9 is a transcription factor in the HMG-box family of genes, and it regulates the expression of genes related to astrocyte development by binding to DNA sequences in regulatory regions of genes to activate or suppress their transcription and expression. Kang et al. [86] showed that Sox9 directly regulates NFIA (nuclear factor-I A) and is necessary for its induction. NFIA and NFIB (nuclear factor-I B) both belong to the NF-I family of transcription factors and are implicated in neuronal development. NFIB interacts with Sox9 to promote astrocyte fate specification [87]. Additionally, zinc finger and BTB domain-containing protein 20 (ZBTB20) has been implicated in regulation of astrocyte cell specification during neocortical development [88]. Recent work by Baranes et al. [89] demonstrated that distinct four TF cassettes (Sox9, NFIA, NFIB, and ZBTB20) can induce human PSCs into astrocyte-like cells with comparable morphology and marker expression, but with subtle differences in their functional properties. Notably, these induced astrocytes displayed variation in cytokine responsiveness, calcium signalling, and maturation state.

Astrocytes are commonly characterised using a panel of molecular markers that capture their developmental state, regional diversity, and functionality. GFAP remains the canonical astrocyte marker, though its expression is largely confined to fibrous and reactive astrocytes, making it insufficient for labelling all subtypes. More pan-astrocytic markers, such as aldehyde dehydrogenase 1 family member L1 (Aldh1L1) and the glutamate transporters GLAST (EAAT1) and GLT-1 (EAAT2), although they are not exclusively astrocytic, provide broader coverage and are frequently used to delineate astrocyte populations with low GFAP expression. Markers of immature astrocytes include nestin, while proteins linked to astrocytic homeostatic functions, such as aquaporin-4 (AQP4) at the perivascular end-feet, and connexin-43 (Cx43) in gap junctions, serve to highlight functionality (astrogliosis/BBB) [90].

### 6.3. Oligodendrocyte Differentiation

Like neurons and astrocytes, OLs arise from the neuroectoderm. In the CNS, OLs develop from oligodendrocyte precursor cells (OPCs) during late embryogenesis, undergoing proliferation, migration, and maturation. Their differentiation stages, gene regulators, and signalling pathways have been defined in vitro [91]. OPCs can be generated from ESC/NPC cultures using factors such as RA, SHH, PDGF, IGF-1, T3, and cAMP [92,93,94]; and are identified by markers including OLIG2, NKX2.2, SOX10, and NG2 [95]. SOX10 upregulation drives the shift from neurogenesis to oligodendrogenesis. Differentiation efficiency varies widely due to culture conditions, with O4 expression ranging from 46% to 90% across protocols. Mouse ESC models are more efficient than human systems, where differentiation takes 5–8 times longer [91]. Direct reprogramming of rodent fibroblasts using OLIG2, SOX10, Ascl1, and NKX2.2 can yield mature OLs within 21 days [96], but these cells show limited myelination and are unsuitable for human therapy due to their xenogeneic nature. Developing xeno-free, faster, and higher-purity methods is essential for clinical application.

Recent protocols differentiate hPSCs into mature OLs through defined stages: hPSCs → neural progenitors (NPs) → OPCs → mature OLs. NPs expressing PAX6 and Nestin mark early OL lineage commitment [97]. NP-to-OPC differentiation relies on neurotrophic and growth factors, such as bFGF, which promotes pre-OPC proliferation and OLIG2 expression while blocking motor neuron fate. Signaling molecules including retinoic acid (RA), sonic hedgehog (SHH), thyroid hormone (T3), and noggin guide OL generation [98]. RA enhances neural/OPC formation [99], and T3 supports pre-OPC survival and OL maturation. Combined SHH, RA, and T3 treatment in early culture halves differentiation time [97].

Due to ethical concerns and limited access to ESCs, iPSCs are now preferred for patient-specific therapies and autologous transplantation, though genetic instability remains a challenge [91]. Wang et al. [100] developed the first hiPSC protocol to generate OLs using feeder-dependent cultures without SMAD inhibitors, achieving ~70% OLIG2/NKX2.2 OPCs in 110–150 days and demonstrating myelination in shiverer mice. Douvaras et al. [99] optimized this to 75 days with ~70% O4 cells by using adherent cultures, RA supplementation, and dual SMAD inhibition. This was achieved by utilising adherent cultures as opposed to suspension cultures as well as RA supplementation and dual SMAD inhibition. Further refinement with Clemastine Fumarate shortened differentiation to 28 days [101].

Recently, TF-based protocols have been developed and decrease culture time to 28 days, and increase efficiency to 70% O4+ oligodendrocyte cells by ectopic expression of SOX10, OLIG2 and NKX6.2 (SON) using lentivirus [16] (Figure 3). Transplantation into shiverer mice also demonstrated the OLs myelinating capacity. Notably, these three TFs in combination have been shown to directly transdifferentiate fibroblasts into oligodendrocyte-like cells in 16 days [102].

OPCs are characterised by their bipolar or tripolar morphology and production of progenitor markers including PDGFaR [92]. Immature premyelinating OLs have a multipolar morphology and express O4 antibody. Typically, the myelinating capacity of terminally differentiated OLs are tested by the transplantation of terminally differentiated OLs into murine models. However, methods fail to develop a pure OL population and the disparity of expression of these specific OL lineage markers in culture is a major issue [96]. Different markers are also expressed across produced OL populations including GalC and O1 [94] and long-term differentiated populations positively express markers MBP, MOG and MAG [103]. The myelinating potential has been demonstrated in vitro by using co-culturing of OLs and neurons or in vivo by transplantation into shiverer mouse models [92]. Demyelination can also be induced by cuprizone, a copper chelator that interferes with complex IV of the mitochondrial respiratory chain as well as with cyanides that block complex IV in the respiratory chain [30]. In addition, cell selective immune mechanisms can cause autoantibodies to target the epitope that is extracellularly located on the OLs causing demyelination by activating the complement pathway or recognising Fc receptors by macrophages [31].

### 6.4. Microglia Differentiation

**Figure 5:**
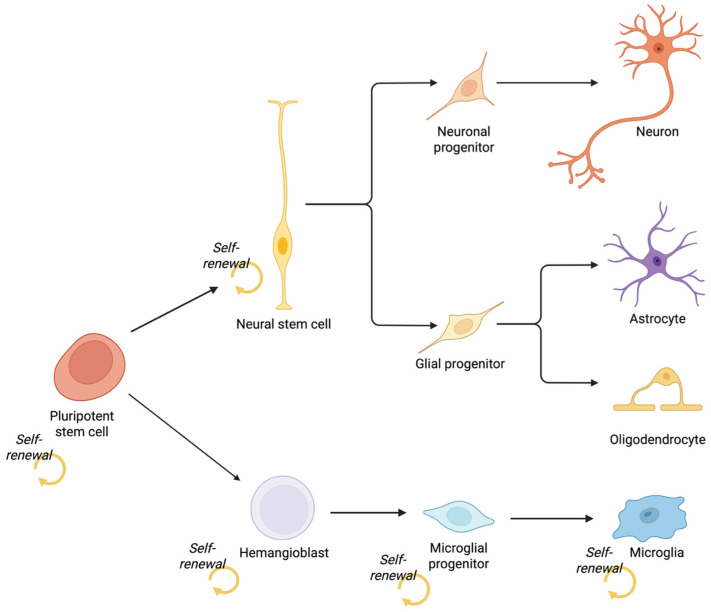
Developmental origins of central nervous system cell types. Pluripotent stem cells give rise to distinct neural and immune lineages through separate germ layers. Neurons, astrocytes, and oligodendrocytes originate from the ectodermal lineage via neural stem cells, which self-renew and generate neuronal and glial progenitors. In contrast, microglia arise from mesoderm-derived hemangioblasts, which produce microglial progenitors that populate the CNS. Created in BioRender. Mcdaid, G. (2025) https://BioRender.com/46pkjlp (accessed on 30 September 2025).

As noted earlier, microglia are the sole innate immune cells that permanently reside within the CNS [32], and unlike other glial and neural cells derived from a neuroectodermal lineage, microglia originate from mesodermal haematopoietic precursors [33] (Figure 5). Microglia have been produced in vitro using a range of methods, with growth factor-driven protocols being among the earliest protocols, guiding PSCs toward a myeloid and microglial lineage through carefully defined cytokine and signalling environments. Microglia induced by growth factors and cytokines such as CSF-1, IL-34, and TGF-β1 used alone produced microglial-like cells; but this process also required many weeks differentiating within culture [104].

In addition to these growth factor-based approaches, transcription factor-mediated reprogramming methods have also been developed to induce microglial identity from PSCs. Spi1 is considered a principal regulator of all myeloid cell determination, which includes microglia. Spi1 expression levels affect microglial transcription and activation, as well as microglial phenotype [105]. Chen et al. [17] showed that overexpression of two genes, Spi1 (also known as PU.1) and CCAAT/enhancer-binding protein α (CEBPA), within iPSCs resulted in the generation of microglia-like cells within 10 days: utilising a lentiviral delivery system to produce tetracycline-inducible expression of these two transcription factors (Figure 3). Many current transcription factor protocols rely on lentiviral vector methods, one approach to avoid lentivirus genome integration is non-viral transduction methods such as Sonn et al. [106]; using a doxycycline-inducible Spi1 expression plasmid. 90 percent of cells produced using this method showed normal microglia function and morphology. While non-viral vectors are advantageous in that they avoid the complications associated with virus production, non-viral gene therapy has a lower efficacy and longevity compared to viral vectors [107]. As with all transcription factor methods, microglial methods also have numerous redundancies, for example, Sonn et al. [106] produced human iPSC-derived microglia using the overexpression of Spi1 only showing the use of CEBPA was not needed. More than 90 percent of cells produced using this method expressed microglia specific cell markers such as P2RY12 and CX3CR1, and they displayed normal microglial functions such as inflammatory responses and phagocytosis.

The differentiation and maturation of induced microglia may be confirmed by measuring CD11b expression, which guarantees that the cells have important immunophenotypic traits of primary microglia [17]. An active or mature microglial state is indicated by high CD11b expression, indicating that these cells are ready to react to signals from the CNS. A cytoskeletal protein unique to macrophages and microglia, ionised calcium-binding adaptor protein-1 (Iba1) is frequently utilised in histological research and may be utilised to measure the phenotypic of microglia. Iba1 is involved in microglia movement, phagocytosis, and membrane ruffling and acts as an actin cross-linking protein [108]. Human monocytes and tissue macrophages exhibit the transmembrane glycoprotein protein CD-68, which is a marker of phagocytic activity [109]. Interesting ways to investigate the macrophage-functionality of microglia can be performed by initiating co-culture models of induced microglia with injured neurons, or by forcing them into an activated or rested state by adding pro-inflammatory substances (such as IFNγ or TNFα) or anti-inflammatory cytokines (IL-4, IL-10) [110].

### 6.5. Comparison with Alternative Reprogramming Strategies

Although TF-based differentiation remains one of the most precise and rapid methods for guiding pluripotent stem cells (PSCs) toward specific neural and glial identities, it is not without limitations. TF-based differentiation relies on the exogenous delivery of lineage-defining TFs, such as Ngn2, ASCL1, DLX2, SOX9, or SOX10, to activate downstream gene networks that drive fate commitment. This approach offers several advantages: it allows direct and efficient conversion to mature cell types, shortens differentiation timelines, and enables the generation of otherwise inaccessible subtypes. However, TF overexpression typically requires integrating viral vectors, which can introduce insertional mutagenesis, variable expression levels, and batch-to-batch inconsistencies [77]. Furthermore, forced TF expression may bypass intermediate developmental stages, leading to incomplete maturation or transcriptional instability in the resulting cells. One alternative to integrating viral vectors, CRISPR/Cas9-mediated insertion of doxycycline-inducible TFs, are an effective and precise method to introduce transgenes to PSCs for the purposes of differentiation. However, one of the main limitations of generating inducible cell lines using CRISPR/Cas9-mediated insertion into a genomic safe harbor locus is the significant amount of time required to establish and validate each individual cell line. This process typically involves multiple steps, including precise genomic targeting, clonal isolation, and thorough characterisation to confirm both correct integration and functional inducibility, all of which can be labour-intensive and time-consuming.

Recent years have seen the emergence of alternative or complementary strategies, including CRISPR activation (CRISPRa)-mediated reprogramming and small molecule-based induction that aim to overcome the reproducibility, safety, and integration challenges associated with conventional TF overexpression. CRISPRa-mediated activation provides a promising, non-integrative alternative that stimulates endogenous transcriptional programs without permanently altering the genome. CRISPRa (CRISPR activation) systems use catalytically inactive dCas9 fused to transcriptional activators to transiently stimulate endogenous TF networks without genomic integration [111,112]. This enables a more efficient and reproducible differentiation process and reduces off-target transcriptional effects. Although CRISPRa-driven differentiation often proceeds more slowly than direct TF overexpression, it offers improved tunability and scalability for high-throughput applications, and it minimises biosafety concerns related to genomic integration.

Small molecule reprogramming provides a complementary strategy for inducing neural cell fates through precise modulation of intracellular signaling and epigenetic states. Without the need for genetic manipulation, defined chemical cocktails can promote neuronal differentiation and functional maturation. In one example, human astrocytes can be directly converted into functional neurons through sequential exposure to a set of nine small molecules that suppress glial programs while activating neuronal pathways [113]. This rapid reprogramming, achieved within 10 days, involves epigenetic remodelling and transcriptional activation of NEUROD1 and Ngn2. The resulting neurons form synaptically connected networks with synchronous activity and survive for extended periods both in vitro and after transplantation in vivo. Although chemical reprogramming offers a cost-effective and non-integrative alternative to TF-based methods, challenges remain in achieving subtype specificity and cellular homogeneity.

## 7. Two-Dimensional Co-Culture Systems

Neuronal monocultures often fail to capture the complexity of the CNS, neuron-glia two-dimensional (2D) co-cultures provide a controlled yet physiologically relevant environment in which glial contributions to neuronal survival, synaptic activity, and homeostasis can be observed. Two-Dimensional culture systems offer advantages such as ease of manipulation, cost-effectiveness, and high reproducibility, while allowing direct observation and controlled study of cell interactions under simplified conditions (Table 1). Unlike neurons, glial cells such as astrocytes and microglia are difficult to study in vitro because they rapidly enter a reactive state after being placed in culture [114]. As a result, their gene expression profiles differ substantially from those observed in glia analysed directly after isolation from primary brain tissue [51].

2D culture systems have become a cornerstone for investigating the electrophysiological properties of excitatory neurons, particularly when coupled with multi-electrode arrays (MEAs) that allow high-resolution monitoring of spontaneous and evoked network activity. In most studies, these neuronal cultures are supported by primary astrocytes, which provide critical trophic factors, promote synapse formation, and help maintain overall network stability. While the inclusion of astrocytes is essential for sustaining neuronal health, it represents only a fraction of the cellular diversity present in the central nervous system. Other glial populations, such as microglia, oligodendrocytes, and OPCs, play distinct and often complementary roles in modulating neuronal excitability, synaptic pruning, myelination, and neuroinflammatory responses [52,53,124,125]. The absence of these cell types in standard 2D cultures limits the ability of these models to fully represent the complex cellular interactions that occur in vivo, potentially reducing the physiological and translational relevance of electrophysiological findings. Therefore, incorporating a broader repertoire of glial and non-neuronal cells into 2D excitatory neuron cultures could yield models that more accurately reflect native network dynamics, offering improved platforms for studying neuronal function, disease mechanisms, and therapeutic interventions. Moreover, the intrinsic functions of glial cells are intimately tied to their role in supporting neurons within the highly organised and dynamic three-dimensional architecture of the CNS [126]. This spatial complexity, which encompasses interactions with neurons, other glia, extracellular matrix components, and vascular elements, is fundamental to the manifestation of physiologically relevant glial properties. Consequently, conventional 2D culture systems fail to emulate these intricate cellular and structural relationships, limiting their utility for modelling the full spectrum of glial biology [51].

## 8. Three-Dimensional Organoids

Three-dimensional (3D) culture systems for CNS cells provide a more physiologically relevant microenvironment than 2D models, enabling the recreation of complex cellular architectures, extracellular matrix interactions, and spatial signalling that more closely mimic in vivo brain tissue.

Many brain organoid models are generated from PSCs that are directed toward a non-specific neuroectodermal fate, giving rise predominantly to excitatory, inhibitory, neuronal and astroglia lineages while lacking contributions from mesodermal progenitors. As a result, these organoids typically do not contain microglia, which arise from yolk sac-derived mesodermal precursors [34,35]. Although organoids can successfully mimic key aspects of neuronal differentiation, synaptogenesis, and cortical layer formation, they often fail to reproduce the immune and homeostatic functions normally mediated by microglia, thereby limiting their ability to fully model processes such as neuroinflammation, synaptic pruning, and responses to injury or disease. Recent efforts to include microglia in cortical organoids from dox-inducible overexpression of Spi1 generated microglia-like cells [120], however a subset of Spi1-overexpressing cells using this method failed to form microglial clusters implying that conversion efficiency may be limited.

A persistent limitation in the development of cerebral organoids is their inadequate vascularisation. In vivo, the extensive vascular network is essential for the delivery of oxygen and nutrients and the removal of metabolic waste, thereby supporting the high energetic demands of neural tissue [127]. In contrast, brain organoids are dependent on passive diffusion, which restricts their growth, viability, and capacity to model later stages of development. Once organoids exceed a few millimetres in diameter, central regions commonly exhibit hypoxia and necrosis, compromising tissue organisation and cellular diversity [128]. Strategies to address this, including the co-culture of endothelial cells by dox-inducible overexpression of ETV2 [121], or transplantation into animal hosts [129,130], have achieved partial success, but none fully address the complexity or dynamic functionality of the cerebrovascular system. The transplantation of brain organoids into animal hosts additionally raises new ethical concerns, particularly regarding animal welfare, the potential for altered cognition or behaviour, and the moral implications of creating human–animal chimeric systems, especially in the context of potentially sentient human organoids [131,132]. The absence of physiologically relevant vascularisation therefore remains a critical barrier to the maturation and translational utility of brain organoid models.

The use of inducible systems to regulate the expression of these defined transcription factors offers a promising strategy for generating brain organoids with greater physiological fidelity. Such approaches could, in principle, allow for the controlled introduction and tuning of specific neuronal and glial subtypes at defined ratios, thereby better mimicking in vivo cellular composition. Furthermore, the same strategy could be extended to incorporate vascularisation elements, providing a more representative microenvironment that supports neuronal-glial function.

3D bioprinting has emerged as a powerful tool for engineering complex tissue constructs by enabling the precise spatial organisation of multiple cell types within 3D architectures. Three-Dimensional bioprinting offers the potential to recreate the complex cellular architecture of the brain, precisely positioning neurons, glia, and supporting cells such as vascular elements to model native neural tissue organisation and function [133,134]. Unlike traditional scaffold-based or self-assembly approaches, bioprinting allows for controlled deposition of cells and biomaterials in anatomically relevant patterns, ensuring that each cell type occupies its physiologically appropriate location and is present in the correct relative proportion [135]. This level of control is critical to exemplify the neural microenvironment.

### The Neurovascular Unit in Organoid Models

The neurovascular unit (NVU) represents a dynamic multicellular complex that coordinates neuronal activity with vascular function to maintain brain homeostasis. It consists of endothelial cells forming the inner lining of cerebral capillaries, pericytes embedded within the vascular basement membrane, astrocytic end-feet that envelop the vasculature [23], microglia that provide immune surveillance [136], and neurons that regulate local blood flow through neurovascular coupling. Together, these elements sustain the integrity of the blood–brain barrier (BBB), regulate metabolic exchange, and mediate responses to injury or inflammation. Dysfunction of the NVU is increasingly recognised as a key contributor to a broad spectrum of CNS disorders, including stroke, AD, and MS. Recreating the NVU (Figure 1) comprising neurons, astrocytes, pericytes, endothelial cells, and microglia is vital for accurately modelling CNS function. Recent advances integrate ETV2- into cerebral organoids [121] to improve perfusion and barrier formation, yielding models that better emulate the blood–brain barrier and vascular coupling.

Transcription factor-based differentiation strategies provide a powerful framework for reconstructing the NVU by enabling the integration of neuronal, glial, vascular, and immune components within organoid systems. Recent studies have demonstrated that fusion of independently induced vessel and brain organoids yields vascularised brain organoids exhibiting robust, self-organised vascular network-like structures that enhance neurogenesis, support the formation of blood–brain barrier (BBB)-like interfaces, and permit the incorporation of functional microglia capable of immune responsiveness and synaptic engulfment [137].

## 9. Current Challenges and Future Directions

Despite the remarkable progress achieved through TF-based differentiation of PSCs, several key challenges continue to limit their widespread application in disease modelling and regenerative medicine. While TF-driven strategies allow rapid and lineage-specific differentiation, they often lack reproducibility and consistency across laboratories and even across populations with broadly non-specific neuronal identities arising from Ngn2 reprogramming [80], PSC lines [138,139], and delivery systems. Variability in TF expression levels and timing can yield heterogeneous populations, undermining the precision these approaches were designed to provide.

Another major limitation lies in the incomplete functional and phenotypic maturation of TF-induced neural cells. Neurons generated by Ngn2, for instance, are neuronal in nature but not restricted to a specific subtype [82] and often exhibit incomplete maturation, while TF-derived astrocytes [89] and oligodendrocytes [16,96] may lack regional identity or myelination capacity comparable to their in vivo counterparts.

The integration of non-neuronal components remains another pressing challenge. Growth factor differentiation from PSCs largely results in ectodermal lineage, resulting in the absence of microglia and vascular structures derived from mesodermal progenitors. These omissions hinder accurate modelling of neuroinflammatory and neurovascular processes that are central to many neurological disorders. Strategies that co-induce endothelial and immune lineages via TFs such as ETV2 for endothelial cells [121] or CEBPA/SPI1 for microglia [17,106], offer promising avenues for building more physiologically complete systems.

Finally, ethical considerations are likely to gain increasing importance as transcription factor-based organoids attain higher levels of biological complexity and functional integration. The development of vascularised and multi-lineage organoids [120,121,137] which more closely mimic the architecture and physiology of native tissues raises novel challenges concerning their appropriate use, oversight, and long-term implications. Moreover, the emergence of organoids exhibiting neural activity or characteristics suggestive of potential sentience [132] further intensifies the ethical debate. Such advances blur the boundary between experimental models and entities with possible moral status, calling for the establishment of robust, transparent, and internationally harmonised guidelines.

## 10. Conclusions

Transcription factor-based differentiation of pluripotent stem cells represents one of the most powerful approaches for generating human-relevant neural systems in vitro. By activating lineage-defining gene networks, TF-based strategies have dramatically accelerated the production of neurons and glia, enabling more controlled exploration of neural development, disease mechanisms, and therapeutic screening. However, the field now faces the critical task of transforming descriptive differentiation into reproducible, standardised, and functionally validated methodologies. The current generation of TF-driven models remains limited by incomplete maturation, variability across PSC lines, and the absence of key mesodermal components such as microglia and vasculature. Addressing these issues will require systematic benchmarking through single-cell and electrophysiological validation, the development of integration-free delivery systems, and the incorporation of supportive microenvironments that enhance long-term functionality. TF-driven differentiation of PSCs has accelerated our ability to construct lineage-specific neural systems and dissect the cellular basis of disease. Advances in neuronal and glial differentiation, together with the development of co-culture and organoid systems, have markedly reshaped the experimental landscape of neuroscience and disease modelling. Defined differentiation protocols now enable the generation of diverse and specific CNS cell types from human pluripotent stem cells, providing access to human-relevant neurons, astrocytes, oligodendrocytes and microglia. However, reproducibility, incomplete maturation, and lack of vascular and immune context remain obstacles. The convergence of TF-based programming with CRISPRa activation, small molecule modulation, and bioengineered organoid systems will define the next phase of in vitro neurobiology; moving toward reproducible, patient-tailored neural reconstruction.

Future directions point toward convergence, merging TF-based induction with small-molecule modulation, and bioengineered organoid or bioprinted platforms. These hybrid systems promise greater precision, safety, and scalability, paving the way for patient-specific modelling and cell-based therapies. As reproducibility and ethical frameworks mature, TF-driven differentiation will not only deepen our understanding of the human brain but also open translational avenues for treating neurodegenerative and neurodevelopmental disorders.

## Figures and Tables

**Table 1:** Comprehensive comparison of 2D in vitro and 3D brain organoid models used in neurodegenerative disease research. This table outlines key examples, major advantages, and critical limitations of each system, emphasising how 2D cultures provide a simple, cost-effective, and high-throughput platform for mechanistic studies and drug screening, while 3D organoids more closely mimic the complex cellular diversity, spatial architecture, and neural network activity of the human brain. Despite their improved physiological relevance, organoids remain limited by challenges such as lack of vascularization, variability between batches, and incomplete maturation, highlighting the complementary roles of both models in advancing understanding and therapeutic development for disorders such as Alzheimer’s, Parkinson’s, and other neurodegenerative diseases.

Model Type	Examples	Advantages	Disadvantages
2D co-culture in vitro models	- Primary neurons (rodent [115] or human [116])	- Simple, well-established - Cost-effective	- Lack 3D architecture and brain-like cytoarchitecture
	- Immortalised cell lines (e.g., SH-SY5Y, PC12 [117,118])	- High-throughput for drug screening and mechanistic studies	- Limited cell–cell and cell–matrix interactions - Poor modelling of long-term aging, vascularisation, and complex circuitry
	- PSC-derived neurons & glia in monolayer culture [119]	- Human iPSCs capture patient-specific mutations [119]	- Over-simplified microenvironment compared to the human brain
3D brain organoids	- Human iPSC-derived cerebral organoids [98] - Human ESC-derived cerebral organoids [120,121]	- Mimic 3D brain structure and layered organisation - Support multiple neural cell types (neurons, astrocytes, oligodendrocytes)	- No full vascularisation → limited nutrient/oxygen diffusion and long-term growth - Lack systemic components (immune system/microglia, blood–brain barrier)
	- Region-specific organoids (e.g.: midbrain for PD, cortical for AD [122,123])	- Capture aspects of human-specific development and disease mechanisms - Allow modelling of network activity and microcircuitry	- High variability between batches and long culture times - Limited maturation and aging - Lower throughput and higher cost vs. 2D

## Data Availability

No new data were created or analyzed in this study. Data sharing is not applicable to this article.
